# 2107. Anti-Biofilm Potential of Virulence Factor EsxB of *Staphylococcus aureus* against its own Sensitive Strain: Molecular Evidences

**DOI:** 10.1093/ofid/ofad500.1731

**Published:** 2023-11-27

**Authors:** Faryal Ashraf, Rukesh Maharjan, Atia tul-Wahab, Iqbal Choudhary

**Affiliations:** International Center for Chemical and Biological Sciences, Karachi, Sindh, Pakistan; H.E.J. Research Institute of Chemistry, International Center for Chemical and Biological Sciences, University of Karachi, Karachi-75270, Pakistan., Karachi, Sindh, Pakistan; a. Dr. Panjwani Center for Molecular Medicine and Drug Research, International Center for Chemical and Biological Sciences, University of Karachi, Karachi-75270, Pakistan., Karachi, Sindh, Pakistan; a. Dr. Panjwani Center for Molecular Medicine and Drug Research, International Center for Chemical and Biological Sciences, University of Karachi, Karachi-75270, Pakistan. b. H.E.J. Research Institute of Chemistry, International Center for Chemical and Biological Sciences, University of Karachi, Karachi-75270, Pakistan., Karachi, Sindh, Pakistan

## Abstract

**Background:**

*Staphylococcus aureus* express different proteins that modulate intracellular bacterial survival and increase antimicrobial resistance with higher mortality rate. The EsxA and EsxB, substrates of the ESAT-6 like secretion system (Ess) are important during intracellular *S.aureus* infection. The genes EsxA and EsxB are present in close proximity with 6 other genes, including EsaA, EssA, EsaB, EssB, EssC, and EsaC.

**Methods:**

EsxB virulence gene from vancomycin resistant *S. aureus* was cloned and transformed into expression cell line E.coli BL21(DE3) cells under IPTG induction. The expressed proteins were purified using affinity and size exclusion chromatography. Purity and protein structures were checked by using NMR spectroscopy and circular dichroism. Further, purified proteins were evaluated for it's hemolytic and antibiofilm property against S. Aureus (ATCC 6538).

**Results:**

he purified EsaB protein showed well spread spectra of 1H- NMR spectrum, however, the 15N, 1H-HSQC spectrum showed overlapped peaks or contours in the region of 6.5-10 ppm indicating the absence of well folded 3D conformation in solution state. Circular dichroism showed the presence of beta sheets in structure of EsxB virulence factor. EsxB protein showed antibiofilm activity against *S.aureus* (ATCC 6538) when treated exogenously. It also showed non hemolytic activity indicating it's non-cytotoxic nature.

**Conclusion:**

This study explains the evidence that bacterial virulent protein EsxB possess antibiofilm activity against its own sensitive strain.

**Disclosures:**

**All Authors**: No reported disclosures

